# Stem cell research and regenerative medicine: meeting report from the Royan International Stem Cell Congress 2025

**DOI:** 10.1186/s13036-026-00702-4

**Published:** 2026-06-04

**Authors:** Niloofar Bajool, Mehdi Abdolazimi, Mohsen Razzaznian, Zeinab Zakerian, Sharif Moradi

**Affiliations:** 1 Department of Stem Cells and Developmental Biology, Cell Science Research Center, Royan Institute for Stem Cell Biology and Technology, ACECR, Tehran, Iran; 2 Department of Stem Cells and Developmental Biology, School of Basic Sciences and Advanced Technologies in Biology, University of Science and Culture, Tehran, Iran

**Keywords:** Stemness, Pluripotency, Organoids, Regenerative medicine, Cell therapy, Bioengineering

## Abstract

The 21st Royan International Stem Cell Congress (3–5 September 2025, Tehran, Iran) convened the global stem cell community to assess the accelerating translation of regenerative medicine from bench to bedside. Over three days, 10 thematic sessions, spanning pluripotency, cancer, organoid technology, molecular biomedicine, artificial intelligence (AI), bioengineering, and clinical translation, provided a comprehensive overview of a field undergoing increasing integration. Key presentations reported recent progress in several areas: the generation of primate and human embryo models for developmental study; new strategies in cross-species reprogramming and exosome-based therapeutics; and the application of AI for patient stratification, drug repurposing, and metabolic modeling. Concurrently, sessions on bioengineering showcased developments in next-generation biomaterials, non-viral gene delivery systems, and scalable microfluidic platforms aimed at enhancing therapeutic safety and manufacturability. The congress also featured critical discussions on regulatory frameworks, bioentrepreneurship, and the ecosystem required to support clinical adoption. Collectively, the meeting underscored a continuing shift toward a multidisciplinary paradigm in which foundational biology, emerging technology, and pragmatic translation converge to advance the development of safe, effective, and accessible stem cell-based therapies worldwide.

## Introduction

**Fig. 1:**
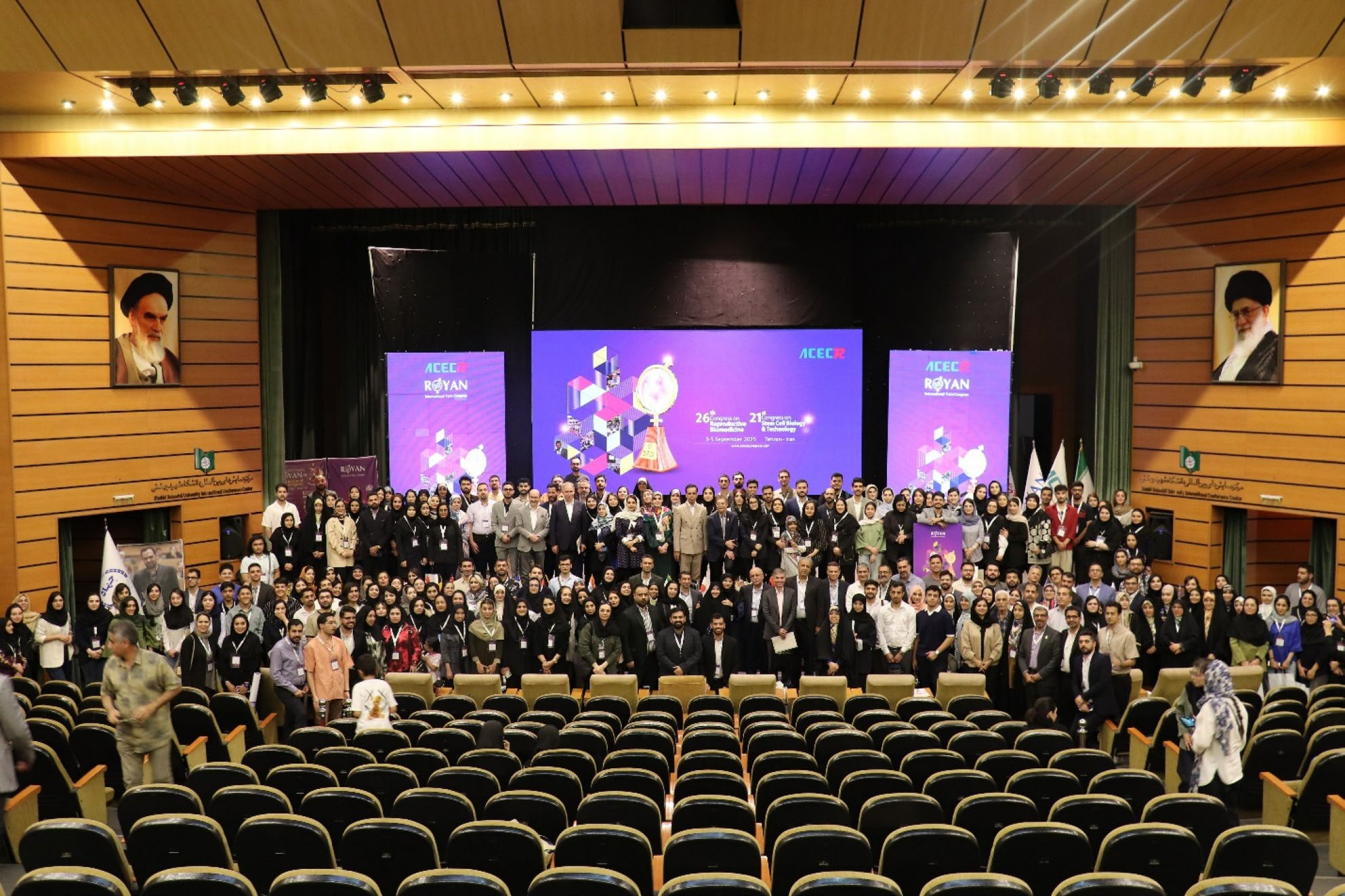
A group photograph of attendees at the 21st Royan International Stem Cell Congress (2025)

Stem cell research stands at the forefront of regenerative medicine, holding huge potential to address fundamental developmental questions and develop novel therapies for a range of incurable diseases. For over two decades, the Royan International Stem Cell Congress has served as a pivotal platform in West Asia, bridging Iranian scientists with global pioneers to accelerate progress in this dynamic field (Fig. 1). Building on a legacy of historic achievements, including the development of the first human embryonic stem cell (ESC) line in the region and advancing natural killer (NK) cell therapies to clinical trials, the congress continues to champion cutting-edge science. The congress welcomed more than 800 participants, of whom 554 (approximately 69%) were women. A detailed breakdown of the 44 invited speakers and panelists, including their gender and leadership status, is provided in Table 1. The congress also featured invited speakers from multiple countries outside Iran, reflecting its international scope. Over its 21-year history, the Royan Stem Cell Congress has fostered numerous international collaborations, co-authorships, and joint publications with scientists worldwide (for example [1–7]). In addition, 25 biotechnology and pharmaceutical companies participated in the congress as exhibitors or sponsors.

**Fig. 2:**
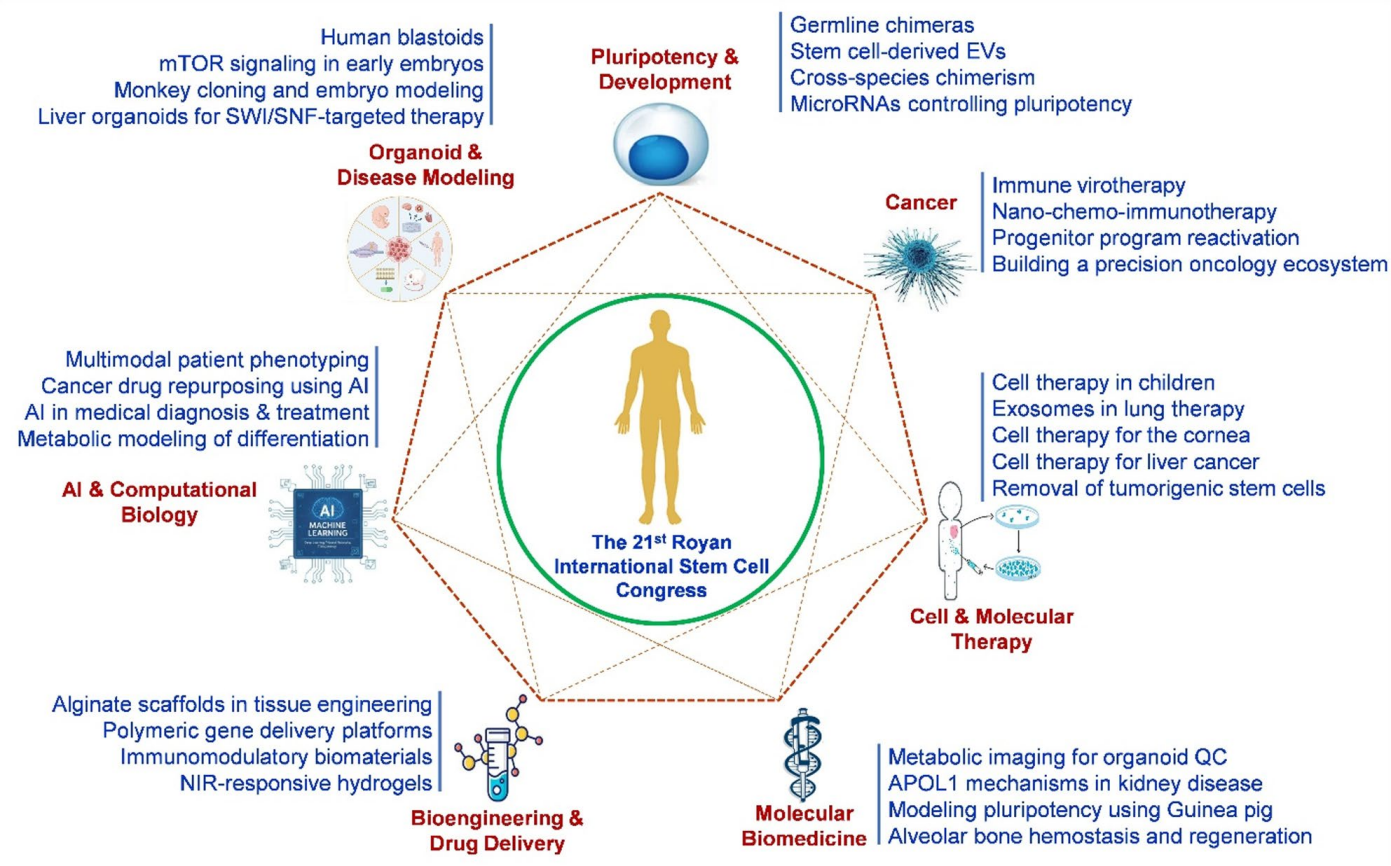
Thematic framework of the 21st Royan International Stem Cell Congress. The scientific program was structured around seven interconnected core themes that define the current trajectory of regenerative medicine. These themes highlight the convergence of foundational biology (Pluripotency & Development, Cell & Molecular Therapy), advanced model systems (Organoid & Disease Modeling), clinical translation (Molecular Biomedicine, Cancer Research), and enabling technologies (AI-Driven Biology, Bioengineering & Drug Delivery)

Several distinctive characteristics make the Royan Stem Cell Congress an attractive hub for international collaboration. Unlike many Western countries, Iran’s Islamic jurisprudence permits research on both ESCs and aborted fetal tissue, offering unique experimental opportunities that are not widely available elsewhere. A nationwide and international network of cord blood banks, alongside clinically approved cell-based products and a growing biotechnology sector, demonstrates a mature translational ecosystem. The congress also benefits from increasing female participation and a large cohort of enthusiastic graduate students who actively seek international placements and collaborations. By intentionally inviting scientists whose expertise addresses specific challenges faced by Royan and other Iranian researchers, the congress facilitates targeted and productive scientific exchange. These features collectively position the Royan Stem Cell Congress as a distinctive and valuable partner for the global stem cell community. An overview of the major scientific themes presented at the 21st Royan International Stem Cell Congress is summarized in Fig. 2.

## Pluripotency and development

The inaugural day of the Royan Stem Cell Congress delved into the fundamental molecular mechanisms governing pluripotent stem cells (PSCs), exploring their profound implications for developmental biology and cellular reprogramming. The session provided novel insights into the principles of pluripotency, ranging from intracellular fate decisions to its translational application across diverse species. Deepa Subramanyam (National Centre for Cell Science, India) elucidated how the intricate balance of endocytosis, secretion, and feedback signaling directs stem cell fate. Her research demonstrated that clathrin heavy chain (CLTC)-mediated endocytosis helps maintain pluripotency by regulating E-cadherin recycling and targeting TGF-β receptor type 1 (TGF-βR1) for lysosomal degradation, whereas caveolin-mediated uptake promotes differentiation. Knockdown of *Cltc* led to loss of pluripotency, reduced E-cadherin levels, and increased TGF-β and ERK signaling. Computational modeling illustrated these signaling pathways as a form of “molecular traffic” [8], highlighting the necessity for precise regulatory control to enhance the efficiency of cellular reprogramming and tissue integration for regenerative medicine.

Pierre Savatier (INSERM, France) subsequently addressed the challenges associated with generating germline-competent chimeras from rabbit PSCs. By reprogramming cells to a naïve state using specific factors, including KLF2, ERAS, and PRMT6, his team achieved robust embryonic integration (reaching up to 100% in specific organs) and confirmed successful germline transmission [9]. These findings establish the rabbit as a highly promising translational model for human disease studies and genetic engineering applications.

**Table 1:** Invited speakers and panelists at the 21st Royan International Stem Cell Congress

Name	Gender	Role at congress	Group leader/Principal investigator?
Deepa Subramanyam	Female	Invited Speaker	Yes
Marzieh Ebrahimi	Female	Panelist/Chair	Yes
Heidar Heidari Khoei	Male	Invited Speaker/Chair	No
Pierre Savateir	Male	Invited Speaker	Yes
Sharif Moradi	Male	Invited Speaker/Chair	Yes
Sara Saeedi	Female	Invited Speaker	No
Jing Liu	Male	Invited Speaker	Yes
Sajad Sahab-Negah	Male	Invited Speaker/Chair	Yes
Seyed Javad Mowla	Male	Invited Speaker/Chair	Yes
Leila Taghiyar	Female	Panelist/Chair	Yes
Sadegh Saghafinia	Male	Invited Speaker/Chair	No
Mehdi Totonchi	Male	Invited Speaker	Yes
Hassan Niknejad	Male	Invited Speaker/Chair	Yes
Fatemeh Salahpour-Anarjan	Female	Oral Presenter	No
Manish Kumar Raikwar	Male	Invited Speaker	Yes
Daria Kuzretsova	Female	Invited Speaker	Yes
Roya Salehi	Female	Invited Speaker/Chair	Yes
James Adjaye	Male	Invited Speaker	Yes
Mehdi Abdolazimi	Male	Oral Presenter	No
Seyed Mahdi Khaliq-Razavi	Male	Invited Speaker/Chair	Yes
Seyed Amir Marashi	Male	Invited Speaker/Chair	Yes
Mohammad-Reza Abolghasemi	Male	Invited Speaker	Yes
Sara Taleahmad	Female	Invited Speaker/Chair	Yes
Mohsen Reza Heidari	Male	Invited Speaker	Yes
Mohammad Hasani-Sadrabadi	Male	Invited Speaker	Yes
Aziz Maleki	Male	Invited Speaker/Chair	Yes
Hamed Daemi	Male	Invited Speaker/Chair	Yes
Fereshteh Karamali	Female	Panelist/Chair	Yes
Majid Ebrahimi Warkiani	Male	Invited Speaker/Chair	Yes
Ensieh Hajizadeh-Saffar	Female	Invited Speaker/Panelist	Yes
Ali Vasheghani-Farahani	Male	Invited Speaker/Panelist	Yes
Hoda Madani	Female	Invited Speaker/Panelist	No
Kamran Mansouri	Male	Invited Speaker	Yes
Massoud Vosough	Male	Invited Speaker/Chair	Yes
Khosrow Jadidi	Male	Invited Speaker/Chair	Yes
Tomo Saric	Male	Invited Speaker	Yes
Elaheh Khodadoust	Female	Invited Speaker	No
Mahsa Mollapour Sisakht	Female	Invited Speaker/Chair	Yes
Laurent David	Male	Invited Speaker	Yes
Zhen Liu	Male	Invited Speaker	Yes
Nasser Aghdami	Male	Invited Speaker/Panelist	Yes
Reza Hassan-Sajedi	Male	Invited Speaker/Panelist	Yes
Mohammad-Javad Rasaee	Male	Invited Speaker/Panelist	Yes
Melika Zamanian	Female	Panelist	No

Further exploring the regulatory landscape, Sharif Moradi (Royan Institute, Iran), who also served as the Scientific Secretary of the congress, emphasized the critical function of microRNAs (miRNAs) in establishing and maintaining ESC pluripotency. Through small RNA sequencing, his group identified several miRNAs, in particular miR-541-5p, miR-410-3p, and miR-381-3p, embedded in the *Dlk1-Dio3* locus as a central regulatory hub supporting pluripotency [2, 3]. Functional assays revealed that specific miRNAs, such as miR-302b-3p, promote ESC derivation and maintenance, while others, including let-7d-3p, act as inhibitors [2]. Interestingly, miR-302b-3p transient overexpression supported the maintenance of mouse ESCs in the absence of leukemia inhibitory factor (LIF) [10]. These results underscore miRNA networks as pivotal determinants of stem cell identity and derivation efficiency. The session continued with a landmark presentation by Jing Liu (Chinese Academy of Sciences, China), who reported the successful derivation of naïve PSCs from non-human primates. These cells demonstrated a remarkable capacity to form viable chimeric monkeys, with donor cell contributions reaching up to 90% across various tissues. Furthermore, his team generated monkey blastoids that progressed to the gastrulation stage in vitro [11]. These breakthroughs provide unprecedented tools for studying primate embryogenesis, advancing genetic engineering, and improving disease modeling. Concluding the session, Sajad Sahab-Negah (Tehran University of Medical Sciences, Iran) shifted the focus to the central nervous system, highlighting the dual role of astrocyte-derived exosomes in brain homeostasis and neurological pathology. He presented evidence that these extracellular vesicles can modulate neuroinflammatory pathways and support tissue repair following injury [12]. animal studies suggest that delivering these exosomes via engineered scaffolds significantly enhances neurogenesis and improves behavioral outcomes, offering a novel therapeutic avenue for conditions such as multiple sclerosis and Alzheimer’s disease.

## Cancer

The scientific program then transitioned to a dedicated session on cancer, shifting the focus to cutting-edge research in oncology. This segment highlighted advances in precision medicine, novel immuno- and nano-therapies, and innovative strategies to understand and treat aggressive malignancies such as glioblastoma (GBM). Seyed Javad Mowla (Tarbiat Modares University, Iran) opened the session by outlining the imperative for a coordinated precision medicine ecosystem in Iran. He introduced a novel national cancer information platform designed to centralize genomic, clinical, and therapeutic data for specialists while providing accessible information for patients. He highlighted foundational initiatives, including the National Cancer Congress for young researchers, support for biotechnology startups, and expanded access to next-generation sequencing as critical drivers for building a robust infrastructure for data integration and innovation. His presentation underscored the necessity of standardized molecular testing, equitable diagnostic access, and strengthened collaboration between academia, industry, and healthcare to improve cancer outcomes.

Subsequently, Mehdi Totonchi (Royan Institute, Iran) presented compelling preclinical data on Onco-VV-TT, a modified vaccinia virus engineered for oncolytic activity. In U251 glioblastoma cells, the virus demonstrated efficient replication and induced substantial cell death, with 3D spheroid assays confirming reduced tumor cell viability and impaired growth. In animal models, Onco-VV-TT suppressed tumor progression, and its combination with NK cells provided even greater therapeutic benefit. Histopathological analysis revealed enhanced necrosis and apoptosis within tumor tissues, accompanied by decreased expression of angiogenesis- and stemness-associated markers, with limited off-target toxicity based on biochemical, hematological, and immunological assessments. This combined approach positions viro-immunotherapy as a highly promising strategy for treating GBM [13]. Next, Sadegh Saghafinia (University College London, UK) provided a conceptual framework for the origins of cancer stem cells (CSCs). He explained that CSCs can emerge from progenitor cells or through the dedifferentiation of tumor cells, processes driven by microenvironmental cues and epigenetic reprogramming. He argued that therapeutic efforts must concurrently target the CSCs and their supportive niche to effectively reduce metastasis and therapy resistance, reframing CSC biology as a dynamic and adaptive system.

Further exploring targeted delivery, Hassan Niknejad (Shahid Beheshti University of Medical Sciences, Iran) compared two paclitaxel delivery systems: human amniotic epithelial stem cell (hAEC)-based carriers versus exosome-mediated nanoparticles. His team demonstrated that hAECs are highly resistant to paclitaxel cytotoxicity over 24 h, with optimal loading and release efficacy at 8,000 ng/ml. While both strategies significantly reduced tumor growth, the stem cell delivery system demonstrated higher potency in vitro and in vivo, including 3.3-fold greater anti-proliferative effects on MCF-7 breast cancer cells and 4.8-fold greater effects on HeLa cervical cancer cells, accompanied by enhanced apoptosis [14]. However, exosome-based delivery exhibited a superior safety profile, highlighting a critical trade-off between therapeutic efficacy and clinical safety in cancer therapy. Concluding the session, Fatemeh Salahpour-Anarjan (Tabriz University of Medical Sciences, Iran) introduced an innovative nano-platform for GBM treatment: cyclophosphamide-loaded chitosan nanoparticles functionalized with targeting peptides. In glioma-bearing rat models, this targeted delivery system induced immunogenic cell death, promoted significant T-cell infiltration into the tumor microenvironment, and substantially prolonged survival with minimal off-target toxicity. These results suggest a powerful new combinatorial nano-chemo-immunotherapy platform with strong potential for clinical translation in GBM treatment.

## Molecular biomedicine

The second day of the congress commenced with the “Molecular Biomedicine” session, which provided a comprehensive overview of the regenerative medicine pipeline. This segment elegantly connected foundational research on novel disease models with tangible therapeutic strategies, building a narrative from biological discovery to clinical application. The session opened with a presentation by Manish Kumar Raikwar (Chinese Academy of Sciences, China), who proposed the genetically underutilized guinea pig as a powerful next-generation model for human disease. His team established guinea pig epiblast stem cells (gpEpiSCs) from post-implantation embryos, confirming their primed pluripotency through the expression of key markers (OCT4, SOX2, NANOG), a stable karyotype, and a dependency on FGF2/ACTIVIN A signaling. Multi-omics analysis revealed a unique transcriptional and epigenetic landscape. Critically, they developed optimized superovulation and embryo transfer protocols to enable transgenic studies. Demonstrating the model’s translational value, AAV9-mediated Gulo gene therapy successfully rescued Vitamin C deficiency in knockout models, restoring survival, neuromuscular function, and metabolic health [15]. This work validates an integrated pipeline from stem cell derivation to in vivo therapeutic intervention.

Building on the theme of innovative models, James Adjaye (University of Düsseldorf, Germany) presented a human urine-derived cellular system to study apolipoprotein L1 (APOL1)-mediated kidney disease. His research identified several upregulated proteins, including ADIPOQ, DPP4, LCN2, RETN, and TNFRSF1A, in the secretome of individuals with high-risk APOL1 variants (G1/G2), with further cytokine array analysis revealing elevated levels of 17 additional inflammatory and fibrosis-associated factors. Using SIX2-positive renal progenitor cells isolated from urine of G1/G1 and G2/G2 individuals of African descent, his team differentiated them into podocytes. Exposure to interferon-gamma (IFN-γ) for 24 h triggered significant APOL1 upregulation and a pronounced cytokine/chemokine response (including CXCL1, CXCL9, CXCL10, CXCL11, CCL20, and others), a pathogenic cascade that was significantly reduced by pre-treatment with the JAK inhibitor Baricitinib at 1–10 µM [16]. Ongoing work to reprogram these patient-specific cells to induced pluripotent stem cells (iPSCs) promises a powerful platform for future mechanistic studies and drug screening. The critical link between model development and clinical manufacturing was emphasized by Daria Kuznetsova (Institute of Oncology and Biomedical Technologies, Russia), who focused on quality control for regenerative products. She demonstrated the use of Fluorescence Lifetime Imaging Microscopy (FLIM) for the non-invasive, label-free metabolic evaluation of human gingival and dental pulp-derived organoids. By analyzing NADH lifetime, her approach identified distinct metabolic profiles for different cell sources and assessed their resilience to bioprinting-associated stressors like UV exposure and thermal variation [17]. This establishes FLIM as a vital tool for pre-implantation quality assurance in 3D bioprinting applications.

Transitioning to direct clinical application, Roya Salehi (Tabriz University of Medical Sciences, Iran) addressed the challenge of post-tooth extraction hemorrhage and bone resorption with a triple-function scaffold exhibiting hemostatic, osteogenic, and angiogenic properties. In vitro assays confirmed suitable hemostatic performance through blood clotting index (BCI) and platelet activation. The biomaterial enhanced the osteogenic differentiation of bone marrow stem cells, elevating RUNX2 and OCN expression, ALP activity, and mineralized matrix deposition. In a rat mandibular model, the scaffold achieved a nearly two-fold reduction in bleeding and promoted bone regeneration, with Micro-CT revealing a bone volume/tissue volume (BV/TV) of 85%, outperforming commercial controls. Concluding the session, Mehdi Abdolazimi (Royan Institute, Iran) delved into the fundamental molecular mechanisms governing pluripotency. His presentation detailed how miR-363-3p promotes the formation of mouse ESCs by precisely modulating the autophagy pathway. The research identified a key autophagy regulator as a target of the miRNA, revealing a novel regulatory axis where controlled autophagy is essential for efficient derivation of ESCs (unpublished data). This work adds a critical layer to our understanding of the metabolic and molecular control of cell fate.

## AI and computational biology

This session was dedicated to the transformative role of AI and computational modeling in biomedicine. Presentations showcased AI-driven frameworks for patient health profiling, personalized medicine, and drug discovery, alongside genome-scale metabolic models (GEMs) for studying cellular differentiation and disease pathways. Seyed Mahdi Khaligh-Razavi (Royan Institute, Iran) introduced a dynamic, multi-layered framework for patient health profiling. This approach begins with low-cost, scalable data sources, such as digital biomarkers and health records, and progressively incorporates more complex data from blood markers, imaging, and genetics. He highlighted how large language models can integrate these disparate data types to enable robust risk stratification, uncover co-morbidity patterns, and support personalized treatment planning. While this framework promises significant gains in efficiency and accessibility, particularly in low-resource settings, Khaligh-Razavi also underscored critical challenges, including data quality, algorithmic bias, privacy, and the need for adaptable regulatory frameworks. Mohammad-Reza Abolghasemi (University of Tehran, Iran) provided a comprehensive overview of AI applications across the healthcare spectrum. His talk covered the impact of AI on medical diagnosis, patient care, and operational efficiency, citing examples from radiology, cancer treatment planning, and the natural language processing of clinical records. He further illustrated these principles with a case study: an AI-powered platform for the continuous monitoring and management of Parkinson’s disease. This system leverages voice analysis and spiral drawing tests to achieve accurate, non-invasive patient screening and personalized care tracking.

Shifting focus to therapeutic discovery, Sara Taleahmad (Royan Institute, Iran) presented an AI-driven strategy to identify novel therapeutic targets and drug candidates for *FLT3*-mutated acute myeloid leukemia (AML), an aggressive blood cancer with high relapse rates. Her team integrated five GEO datasets comparing 50 wild-type versus 48 FLT3-mutated AML samples, identifying approximately 200 hub genes. Key dysregulated genes included down-regulated CTNNB1, UBB, BIRC5, and FOXO3, and up-regulated JUN, EP300, TP53, FOS, and MAPK14, primarily involved in CCKR and MAPK signaling pathways. JUN, IRS2, and YWHAE were pinpointed as potential therapeutic targets. Subsequently, a virtual screening of FDA-approved drugs identified Venetoclax, Itraconazole, Lutein, and Ponatinib with high binding affinity to YWHAE, while metformin, insulin, and irbesartan were proposed for repurposing (unpublished data), demonstrating AI’s potential to rapidly repurpose existing drugs for resistant cancer subtypes. Concluding the session, Seyed Amir Marashi (University of Tehran, Iran) explored the utility of GEMs in understanding cell fate decisions. He argued that metabolism is not merely a reactive process but an active regulator of cell differentiation, with metabolic intermediates directly influencing gene expression and epigenetic states. GEMs, which integrate genomic and biochemical data, allow for the simulation of metabolic fluxes via flux balance analysis. Marashi presented case studies where GEMs revealed critical metabolic shifts during stem cell differentiation and embryogenesis, enabling the identification of disease-related pathways and therapeutic targets. While acknowledging that GEMs are scalable and mechanistically interpretable, he noted their dependency on accurate manual curation (unpublished data). He concluded by highlighting that emerging hybrid approaches, which combine GEMs with machine learning, offer a promising path toward capturing non-linear, data-driven metabolic behaviors with enhanced predictive power.

## Meet the editors

A dedicated “Meet the Editors” session provided invaluable guidance on navigating the scholarly publishing landscape. Moderated by Massoud Vosough (Royan Institute, Iran), the panel featured insights from Payam Kabiri (Tehran University of Medical Sciences, Iran) and Mohsin Reza Heidari (Baqiatallah University of Medical Sciences, Iran), who detailed editorial processes and the standards required for successful publication. The editors outlined best practices for manuscript preparation, peer review, and enhancing the visibility of published work, consistently emphasizing that innovation, methodological rigor, and strict ethical conduct are the foundational pillars of high-impact science. A significant portion of the discussion focused on the responsible use of AI in research. The panel issued a clear recommendation: AI tools are acceptable for language polishing and editing, but any use of AI for content generation or data analysis must be explicitly disclosed in the manuscript. The conversation also tackled emerging challenges, including the reliability of AI-detection software, the potential for embedded algorithmic biases, and the importance of thoroughly revising and ‘humanizing’ AI-generated text to ensure it accurately reflects the authors’ scientific voice and critical analysis.

Finally, the speakers underscored the importance of integrating scientific writing, research integrity, and ethical AI use into graduate education. They argued that developing strong critical thinking skills and English proficiency is essential for young researchers to make meaningful contributions to the global scientific community. The overarching consensus was that AI should be used as a tool to augment, not replace, human intellect and scholarly effort. In summary, the session provided a clear and practical roadmap for researchers aiming to produce high-quality, ethical, and influential biomedical research.

## Bioengineering and drug delivery

The afternoon session on “Bioengineering and Drug Delivery” showcased groundbreaking advancements at the intersection of materials science, biotechnology, and medicine. The presentations highlighted innovations in polymeric gene carriers, immunomodulatory biomaterials, smart hydrogels, and microfluidic systems, underscoring a collective push toward more sophisticated therapeutic delivery and regenerative strategies. Sara Saeedi (Iran University of Medical Sciences, Iran) opened the session with a novel solution to the classic toxicity, i.e. efficiency trade-off in non-viral gene delivery. His team developed ATP-responsive nanoparticles based on polyethylenimine (PEI) coordinated with zinc ligands. These particles effectively condensed plasmids at low ratios and released their genetic payload in ATP-rich intracellular environments while demonstrating low cytotoxicity. A structurally optimized version (PEI-PBA-Ligand-Zn) achieved superior transfection efficiency compared to standard PEI, an observation attributed to enhanced zinc coordination and proton sponge activity [18].

Building on the theme of advanced biomaterials, Mohammad Hasani-Sadrabadi (University of California [UCLA], USA) presented an implantable scaffold designed to locally recruit and activate T cells in vivo, offering a streamlined alternative to costly ex vivo cell therapies. The biomaterial mimics lymph node properties by delivering immune-activating cues and antibodies while simultaneously inhibiting regulatory T-cell (Treg) activity via TGF-β blockade. In a murine model of aggressive breast cancer, this platform achieved a 40% complete response rate, reduced metastasis, and significantly extended survival. Notably, surviving animals developed protective immunological memory, preventing tumor recurrence upon rechallenge (unpublished data). This strategy demonstrates the potential of bio-inspired biomaterials to induce potent and durable systemic anti-tumor immunity while reducing treatment complexity and cost. Aziz Maleki (Zanjan University of Medical Sciences, Iran) then introduced an injectable, multifunctional hydrogel for infected wound healing. The Alg-HA-Zn-PDA system integrates near-infrared photothermal therapy—generating local temperatures above 50 °C under NIR irradiation—with inherent antibacterial, antioxidant, and hemostatic properties, all while maintaining high biocompatibility. The hydrogel’s three-dimensional porous structure absorbs large quantities of water, providing a moist wound environment and enabling filling of irregularly shaped wounds. In vitro and animal studies confirmed complete bacterial eradication via physical thermal destruction, accelerated wound closure, reduced inflammation, and robust collagen deposition. Treated animals exhibited full epithelialization and hair follicle regeneration [19]. Promising results from early human testing further support the strong commercial potential of these hydrogels as advanced wound dressings that simultaneously control infection and promote tissue repair.

Hamed Daemi (Royan Institute, Iran) presented work on sulfated alginate, a modified biomaterial that combines enhanced solubility and bioactivity with a structural resemblance to native glycosaminoglycans. He demonstrated the versatility of sulfated alginate in forms including hydrogels, nanofibers, and microparticles, all of which exhibited strong cytocompatibility, pro-angiogenic effects, and tissue regenerative capacity. Animal models confirmed accelerated wound healing, improved vascular graft integration, enhanced cartilage repair, and reduced fibrosis. The material also facilitated the delivery of cationic therapeutics, highlighting sulfated alginate as a low-cost, renewable biomaterial for diverse applications in soft tissue engineering [20].

Concluding the session, Majid Ebrahimi Warkiani (University Technology Sydney, Australia) highlighted the pivotal role of microfluidic platforms in advancing precision medicine and biomanufacturing. His team’s innovations included devices for isolating circulating tumor cells (CTCs) and extracellular vesicles (EVs) for liquid biopsy, single-cell chips for metabolic assays, and systems for improving CAR-T production and CRISPR/Cas9 delivery efficiency. He also introduced SMART microcarriers that enable a 20-fold cell expansion with reduced cost and spatial footprint [21]. Looking forward, Warkiani emphasized the future integration of AI-driven predictive models and multi-biomarker cartridges to further enhance the capabilities of these transformative technologies.

## Regulation and standardization

A critical session on “Regulation and Standardization,” chaired by Melika Zamanian (Royan Institute, Iran), provided a comprehensive overview of the regulatory landscape governing advanced therapies in Iran. The panel brought together experts to discuss the legal and practical frameworks essential for translating cellular and gene-based products from the laboratory to the clinic. Ensieh Hajizadeh (Royan Institute, Iran) outlined the key legal considerations in the production and quality control of cellular products. This was complemented by a detailed explanation from Mohammadreza Abedi (Iran Food and Drug Administration, Iran) on the national registration process and regulatory oversight for cell and gene therapies. Further discussions by Ali Vasheghani-Farahani (Tehran University of Medical Sciences, Iran) and Hoda Madani (Royan Institute, Iran) addressed the supervision of private plasmapheresis centers and the critical aspects of designing and conducting clinical trials for advanced therapy medicinal products (ATMPs), respectively.

The panel highlighted Iran’s tangible progress in this field, noting several approved cell-based treatments for conditions such as vitiligo, graft-versus-host disease (GVHD), and chronic wounds. However, significant challenges persist, including complex trial designs, limited patient recruitment, stringent regulatory requirements, high manufacturing costs, and varying levels of acceptance within the physician community. To address these hurdles, the panel proposed several strategic solutions. These included the adoption of adaptive trial designs, the use of real-world evidence as control data, multi-center collaborations to enhance patient recruitment, and leveraging contract development and manufacturing organizations (CDMOs) to streamline production. Innovative reimbursement models, such as installment payments or outcome-based pricing, were also suggested to improve financial sustainability. The session concluded by emphasizing that the successful integration of these advanced therapies into standard care is contingent upon continuous clinician education and transparent communication with patients.

## Cell and molecular therapy

The session on “Cell and Molecular Therapy” commenced with a conceptual framework for regenerative medicine, categorizing current strategies into three main approaches: replacement (direct tissue or organ transplantation), engraftment (integration and differentiation of grafted stem cells), and regeneration (stimulating the body’s innate repair mechanisms). A pivotal theme emerged from recent clinical evidence: most transplanted stem cells exhibit short-term survival, suggesting their primary therapeutic effect is not due to permanent engraftment but rather their paracrine activity. This involves the release of a secretome rich in bioactive molecules and EVs that modulate inflammation and promote endogenous healing. This paradigm shift underscores the potential of EV-based therapies as a potent, cell-free alternative. Kamran Mansouri (Kermanshah University of Medical Sciences, Iran) provided clinical support for this concept. He reported that human placental mesenchymal stem cell (MSC)-derived small extracellular vesicles (hPMSC-sEVs) in severe COVID-19 patients led to significantly reduced mortality (19.04% vs. 54.16% in controls, *P* = 0.015) and longer time to death (28.06 vs. 11.10 days, *P* < 0.001), with a favorable safety profile. Furthermore, a pilot study in 10 patients with systemic sclerosis-associated interstitial lung disease (SSc-ILD) demonstrated significant improvements in 6-minute walk test (from 187 to 229 m, *P* = 0.018) and quality of life (SF-36 score from 88.30 to 91.40, *P* = 0.015) following five consecutive daily intravenous doses of hPMSC-sEVs, with no adverse events reported [22]. These findings position EV therapy as a promising, safe, and scalable regenerative treatment, though the presentation emphasized the necessity for larger, controlled trials to confirm efficacy and standardize clinical protocols.

Shifting focus to oncology, Massoud Vosough (Royan Institute, Iran) presented a research program on hepatocellular carcinoma grounded in evolutionary biology. His team is using a strategy of therapeutic differentiation, which uses evolutionary cues and natural compounds, such as all-trans retinoic acid and conjugated linoleic acid, to re-differentiate cancer cells. This process renders the tumor cells more susceptible to immune attack, chemotherapy, and apoptosis. Preclinical studies showed that when these differentiating agents are combined with NK cells, a significant reduction in tumor growth is achieved (unpublished data). This approach represents a complementary strategy to immunotherapy aimed at overcoming resistance and preventing recurrence.

Khosrow Jadidi (Bina Eye Hospital, Iran) then detailed their cell-based and tissue-engineering methods for corneal regeneration, particularly for keratoconus. His work includes cultivating corneal stem cells on topographic polymers to induce keratocyte-like cells, demonstrating the long-term stability of amniotic membrane grafts without rejection, and utilizing decellularized fish corneas and SMILE-extracted lenticules as low-antigenicity scaffolds. He also introduced a synthetic ring for advanced keratoconus that enables precise femtosecond-laser implantation [23]. These approaches have shown great integration, transparency, and safety, offering scalable solutions for corneal diseases where donor tissue is scarce. Tomo Šarić (University of Cologne, Germany) addressed a critical safety concern in PSC therapies: the risk of teratoma formation from residual undifferentiated cells [24]. He presented salicylic diamines (SM2, SM6, and SM8) that selectively eliminate PSCs from differentiated populations without adversely affecting mature cardiomyocytes. Half-maximal inhibitory concentrations (IC₅₀) for SM2 and SM6 were, respectively, 9- and 18-fold higher for human than murine PSCs. Among them, SM6 emerged as the most promising candidate, significantly reducing PSC and non-PSC contamination in murine embryoid body cultures and enriching cardiomyocyte populations without genetic modification. All tested compounds exerted their toxicity by inhibiting oxygen consumption rate (OCR) in PSCs, with no or only minimal and reversible effects on cardiomyocyte function, including sarcomeric integrity, beating frequency, and ROS levels. Teratoma formation from SM6-treated PSC-derived cardiomyocytes was abolished or delayed compared to untreated cells [25]. His team is further elucidating the mitochondrial mechanisms of action, paving the way for clinical applications to enhance the safety profile of cell-based products. Concluding the session, Elham Khodadoust (Royan Stem Cell Technology, Iran) discussed the application of cell therapy for pediatric neurological disorders, including cerebral palsy (CP) and autism spectrum disorder (ASD). Clinical trials using allogeneic mesenchymal stem cells have demonstrated an excellent safety profile and significant functional improvements in motor skills and social behavior [26]. The therapy is believed to modulate neuroinflammation and support brain connectivity, with therapeutic effects often becoming more pronounced over several months. Khodadoust stressed that continued research is essential to optimize dosing, protocols, and treatment frequency for broader clinical application.

## Organoid and disease modeling

The “Organoid and Disease Modeling” session highlighted the power of stem cell-derived models to unravel developmental mechanisms and disease processes. Presentations highlighted cutting-edge advances in embryonic modeling, cellular signaling, and the growing therapeutic applications of organoid technology. The session opened with a focus on translational output. Mahsa Mollapour Sisakht (Tehran University of Medical Sciences, Iran) addressed the manufacturing challenges of MSC therapies by presenting a cell-free approach. Her work detailed a bioreactor-based system for scalable 3D culture of adipose-derived MSCs (AD-MSCs), which formed compact spheroids by day 5 and secreted a conditioned medium (CM) enriched with higher levels of VEGF, PDGF, FGF2, IGF-1, TGF-β1, and HGF compared to conventional 2D cultures. The AD-MSCs were characterized according to ISCT criteria: adherence to plastic, expression of CD90⁺ and CD105⁺, absence of CD34⁻ and CD31⁻, and confirmed trilineage differentiation potential (unpublished data). This method establishes a robust, standardized platform for generating a potent cell-free therapeutic, offering a pragmatic path for clinical translation.

The focus then shifted to the earliest stages of human development. Heidar Heidari Khoei (IMBA, Austria) utilized human blastoids to investigate the critical peri-implantation period. Through integrated transcriptomic and phosphoproteomic analyses, his team identified the nutrient-sensing mTOR pathway as a master regulator of cell fate decisions. By modulating mTOR activity, they could directly manipulate lineage specification within the blastoids, providing fundamental insights into how metabolic signaling guides human embryogenesis and offering a new model to study the causes of implantation failure [27]. The critical importance of benchmarking these sophisticated models was emphasized by Laurent David (INSERM, France). He presented a biologically curated transcriptomic atlas of human embryos spanning 3 to 14 days post-fertilization (d.p.f.), serving as an essential community resource. Through weighted gene co-expression network analysis, his analysis delineated lineage-specific gene modules and identified key transcriptional regulators, such as ZIC3 and ZSCAN10, driving the naïve-to-primed pluripotency transition. Using SCENIC for gene regulatory network (GRN) inference, his team substantiated the developmental staging of stem cell models via proteomic data, validating that human trophoblast stem cells correspond to an 8 d.p.f. embryo and demonstrating that two extended-culture blastoid models accurately recapitulate key molecular features of peri-implantation embryos. This integrated resource, including scRNA-seq, GRN inference, and immunofluorescence, is browsable through an interactive interface, providing a crucial benchmark for assessing model fidelity (unpublished data).

Finally, Zhen Liu (Chinese Academy of Sciences, China) presented groundbreaking work that pushes the boundaries of in vivo chimerism in primates. His team achieved a technical breakthrough by systematically optimizing culture conditions to generate naïve PSCs in monkeys. This culminated in the birth of a live chimeric monkey with a high donor cell contribution (up to 90%) across diverse tissues, including the germline. Furthermore, he reported the first successful generation of monkey blastoids, which were cultured in vitro to day 17, exhibiting gastrulation and germ layer differentiation [28]. This research establishes a vital, physiologically relevant primate model for evaluating the safety and efficacy of regenerative therapies and for fundamental studies of human development.

## Meet the entrepreneurs

The “Meet the Entrepreneurs” panel provided a critical perspective on the landscape of biotechnology entrepreneurship in Iran. Chaired by Reza Moghadasali (Royan Institute, Iran) and featuring experts including Nasser Aghdami (Royan Institute, Iran), Reza Hasan Sajedi (Tarbiat Modares University, Iran), Mohammad-Javad Rasaee (Rojan Azma, Iran), and Ahmad Hosseini (Erfan Niayesh Hospital, Iran), the session offered guidance for students and early-career researchers seeking to transition into the biotechnology industry. A central theme of the discussion was the distinct skill set required for entrepreneurial success. The panelists emphasized that while a deep personal interest in science is foundational, it must be coupled with robust management and business expertise to build a viable venture. They argued that the core of a successful biotech company lies in its ability to identify a pressing societal need and develop a strategic, market-oriented response. The development of cell therapy in Iran was presented as a seminal case study. What originated as an academic research initiative evolved into a widely adopted clinical treatment through deliberate market development. This success was attributed to a focused strategy of addressing clear patient needs with a therapy that offered improved efficacy at a reasonable cost, demonstrating an effective pathway for translating scientific discovery into tangible public health impact.

## Conclusion and future perspectives

The 21st Royan International Stem Cell Congress served as a definitive snapshot of a field in rapid translation. Across the 10 thematic sessions, three overarching insights emerged that transcend individual presentations. First, the paracentric shift is now clinically validated. Multiple independent studies, from Mansouri’s EV therapy in COVID-19 and SSc-ILD patients (19% vs. 54% mortality, *P* = 0.015) to Niknejad’s comparison of stem cell versus exosome delivery, collectively confirm that therapeutic benefits often derive from secreted factors rather than long-term engraftment. This convergence of clinical and preclinical data positions cell-free platforms as a viable, scalable alternative to live-cell therapies.

Second, enabling technologies are moving from periphery to core. Taleahmad’s AI-driven drug repurposing (identifying Venetoclax and metformin for FLT3-AML), Šarić’s small-molecule PSC elimination (9- to 18-fold IC₅₀ differences), and Mollapour Sisakht’s bioreactor-based cardiomyocyte production (enriched for VEGF, PDGF, FGF2, IGF-1, TGF-β1, and HGF) each demonstrate that computational, chemical, and bioengineering tools are no longer adjuncts but essential drivers of progress.

Third, quantitative benchmarking is maturing the field. David’s transcriptomic atlas (3–14 d.p.f.) and blastoid validation, Liu’s chimeric monkey model (up to 90% donor contribution), and Moradi’s miRNA network mapping (miR-541-5p, miR-410-3p, miR-381-3p at the *Dlk1-Dio3* locus) collectively illustrate a field that is rigorously defining reference standards, measuring efficiency, and validating models against in vivo gold standards.

Emerging directions such as extracellular vesicle-based therapies, organoid-driven disease modeling, AI-assisted therapeutic discovery, and advanced biomaterial engineering are rapidly reshaping translational medicine. Notably, Iranian research groups contributed significantly to these trends, with clinically applied cell-based products, a growing biotechnology ecosystem, and increasing capacity for international collaboration.

However, the path to widespread clinical integration is not without hurdles. Dedicated sessions on regulation and entrepreneurship underscored persistent challenges: complex trial designs, high costs, and specialized infrastructure needs. In response, the congress proposed pragmatic solutions, including adaptive clinical trials, real-world evidence, public-private partnerships, and innovative reimbursement models.

In summary, the 2025 Royan Stem Cell Congress illuminated a clear trajectory. The convergence of quantitative clinical data, rigorous model benchmarking (transcriptomic atlases, chimeric rates), and strategic infrastructure development (bioreactor platforms, regulatory frameworks) is creating a powerful synergy. This collaborative ecosystem, championed by the Royan Institute and its international partners, is paving the way for a future in which cell-, gene-, and tissue-based therapies become routine and accessible. As a meeting report, this article provides a selective overview; some findings described may represent preliminary data requiring further validation.

## Data Availability

No datasets were generated or analysed during the current study.

## Abbreviations

AI: Artificial intelligence
ESC: Embryonic stem cell
NK cell: Natural killer cell
PSC: Pluripotent stem cell
miRNA: MicroRNA
GBM: Glioblastoma multiform
CSC: Cancer stem cell
APOL1: Apolipoprotein L1
IFN-γ: Interferon-gamma
iPSCs: Induced pluripotent stem cell
FLIM: Fluorescence Lifetime Imaging Microscopy
BCI: Blood clotting index
GEM: Genome-scale metabolic model
PEI: Polyethylenimine
Treg: Regulatory T-cell
CTC: Circulating tumor cell
EV: Extracellular vesicle
ATMP: Advanced therapy medicinal product
GVHD: Graft-versus-host disease
CDMOs: Contract development and manufacturing organizations
